# Anti-N-methyl-D-aspartate receptor encephalitis with an imaging-invisible ovarian teratoma: a case report

**DOI:** 10.1186/s13256-016-1067-4

**Published:** 2016-10-24

**Authors:** Zainab M. Abdul-Rahman, Peter K. Panegyres, Margareta Roeck, David Hawkins, Jude Bharath, Paul Grolman, Cliffe Neppe, David Palmer

**Affiliations:** 1 Department of Medicine, Joondalup Health Campus, Joondalup, Western Australia Australia; 2 Academy of Neurology, Joondalup Health Campus, Joondalup, Western Australia Australia; 3 Neurodegenerative Disorders Research Pty Ltd, 4 Lawrence Avenue, West Perth, Western Australia 6005 Australia; 4 Department of Medicine, The University of Western Australia, Perth, Western Australia Australia; 5 Ramsay Health, Joondalup Health Campus, Joondalup, Western Australia Australia; 6 Western Diagnostic Pathology, Joondalup Health Campus, Joondalup, Western Australia Australia

**Keywords:** Encephalitis, Paraneoplastic, NMDA receptor, Teratoma, Oophorectomy, Case report

## Abstract

**Background:**

Anti-N-methyl-D-aspartate receptor encephalitis is a recently discovered disease entity of paraneoplastic limbic encephalitis. It largely affects young women and is often associated with an ovarian teratoma. It is a serious yet treatable condition if diagnosed early. Its remedy involves immunotherapy and surgical removal of the teratoma of the ovaries. This case of anti-N-methyl-D-aspartate receptor encephalitis involves an early surgical intervention with bilateral oophorectomy, despite negative imaging evidence of a teratoma.

**Case presentation:**

A 25-year-old white woman with anti-N-methyl-D-aspartate receptor encephalitis presented with behavioral changes and seizures that were confirmed to be secondary to anti-N-methyl-D-aspartate receptor encephalitis. She required an admission to our intensive care unit for ventilator support and received a number of immunological therapies. Multiple imaging investigations showed no evidence of an ovarian teratoma; she had a bilateral oophorectomy 29 days after admission. Ovarian histology confirmed the presence of a teratoma with neuronal cells. A few days after the operation she began to show signs of improvement and, apart from mild short-term memory loss, she returned to normal function.

**Conclusions:**

Our patient is an example of teratoma-associated anti-N-methyl-D-aspartate receptor encephalitis, in which the teratoma was identified only microscopically. Her case highlights that even with negative imaging evidence of a teratoma, ovarian pathology should still be considered and explored.

## Background

Anti-N-methyl-D-aspartate receptor (anti-NMDAR) encephalitis is a relatively recently identified disease entity of paraneoplastic limbic encephalitis, first described by Dalmau *et al*. [1, 2]. Although the incidence of this disorder is still unknown [3], there has been recent interest in the recognition of immune-mediated central nervous system disorders including anti-NMDAR encephalitis [4].

In two large multicenter studies of herpes simplex encephalitis (HSE) mimics, Whitley *et al*. looked at 432 patients with encephalitis in the period 1973 to 1988 and found that the etiologies of the encephalitis that was experienced by 45 % of the patients were due to herpes simplex virus (HSV), 22 % were due to non-HSV etiologies and 33 % remained without a diagnosis [5]. Chow *et al*. examined 251 patients with encephalitis in the period 1998 to 2012 and concluded that 24 % of cases were due to HSV and 35 % were due to non-HSV encephalitis, including other infections (bacterial, fungal abscess, mycoplasma, varicella zoster virus, tuberculosis) and non-infectious causes such as vasculitis, malignancy, and anti-NMDAR encephalitis [4]. A substantial number of non-HSV etiologies, such as anti-NMDAR encephalitis, were identified in the Chow *et al*. cohort [4], but not in the Whitley *et al*. cohort [5].

Anti-NMDAR encephalitis is a serious yet treatable condition if recognized and treated early [3]. In almost 80 % of cases young females are affected [6]. Patients with this disorder present with rapidly progressive neuropsychiatric symptoms including behavioral disturbance, psychosis, memory deficits, and seizures at onset; patients’ symptoms progress to dyskinesia, autonomic instability, hypoventilation, and coma [1–3].

In 58 % of cases an associated ovarian teratoma is identified [7]. The teratoma contains neuronal cells that result in immunologic sensitization against the NMDA receptors [1, 2, 7]. NMDA receptors are present in high density in the frontotemporal region of the brain and are named after their selective agonist N-methyl-D-aspartate [8, 9]. They are involved in a number of cognitive processes including behavior, memory, learning, and synaptic spasticity [9].

It has been shown that early diagnosis and management is a critical prognostic factor in anti-NMDAR encephalitis [1, 2, 6]. Optimal management of this disorder involves a multidisciplinary team, and the use of aggressive immunotherapy, chemotherapy, surgical removal of the ovarian teratoma, and intensive care unit (ICU) support for hypoventilation and autonomic instability [10]. In 75 % of cases, patients achieve a full recovery or are left with mild deficits; in 25 % of cases, severe residual deficits or death occurs [1–3, 11]. The risk of relapse is reported to be 12 %, which is higher in cases when the tumor is not detected [10]. The mortality rate of this condition is 7 % at 2 years, and is usually secondary to neurological or autonomic dysfunction [6, 10].

**Fig. 1:**
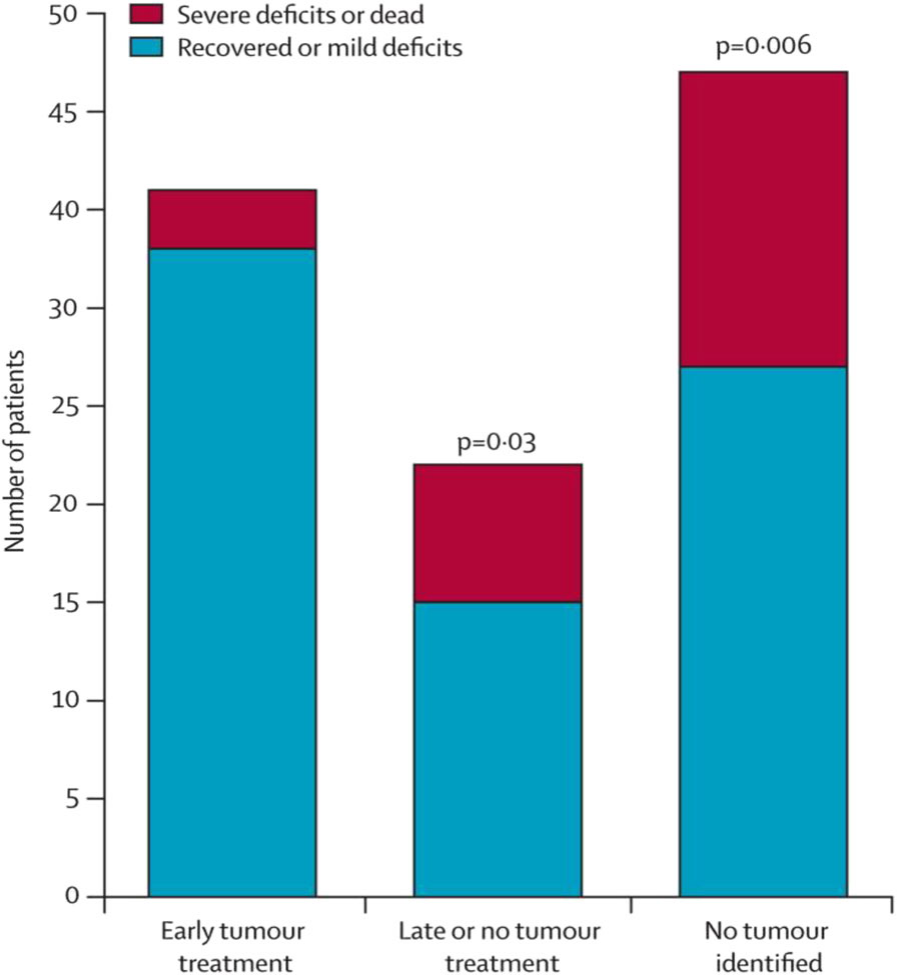
Neurological outcome following tumor removal

In a systemic review of 100 cases of anti-NMDAR encephalitis, early surgical removal of the teratoma with immunotherapy (within 4 months of symptoms onset) showed a better neurological outcome, a lower chance of relapse, and a reduced time to recovery than late or no tumor treatment [11] (Fig. 1).

Acién *et al*., in their systematic review of 174 cases of anti-NMDAR encephalitis between the years of 2007 and 2013, reported that even small teratomas containing nervous tissue may result in severe complications secondary to anti-NMDAR encephalitis and, given these might be difficult to diagnose, it is very likely that prompt intervention is necessary [12]. Although an exploratory laparotomy has been suggested when a teratoma is not identified by imaging, the benefit of this procedure remains uncertain [12]. A case series review by Dalmau *et al*. showed that out of seven patients who underwent exploratory laparotomy, only one case was found to have a teratoma [11].

It has also been described that the teratoma might only be detected several years following the presentation of anti-NMDAR encephalitis [10], or it may only be identified microscopically following oophorectomy or at autopsy several months after admission [12]. However, only a few cases of patients with imaging-negative ovarian teratoma who have had a life-saving oophorectomy have been reported, and for this reason we present our patient.

## Case presentation

A 25-year-old previously well white woman presented with two generalized tonic–clonic seizures, which were preceded by a 2-week history of prodromal flu-like symptoms and nonspecific headache. She was agitated in the emergency department for which she required midazolam.

Her routine bloods were unremarkable. A computed tomography (CT) scan of her brain showed mild generalized cerebral edema and a magnetic resonance imaging (MRI) showed bilateral temporal lobe lesions involving her hippocampi and amygdala, which were more extensive on the right; this is an appearance in keeping with limbic encephalitis. An electroencephalogram (EEG) showed periodic lateralized epileptiform discharges and slow wave changes, which were maximal in the frontal and temporal regions.

She was initially treated with acyclovir for possible HSV encephalitis. She also received levetiracetam to prevent seizures. However, she deteriorated over the next week with behavioral changes, memory impairment, hypoventilation, and coma. She was admitted to ICU 9 days after admission for airway support.

Investigations revealed that her cerebrospinal fluids (CSF) had an elevated lymphocyte count and anti-NMDAR antibodies were strongly positive. Anti-NMDAR antibodies were also detected in her serum. The diagnosis of anti-NMDAR encephalitis was made 11 days after admission. She was commenced on a 5-day course of methylprednisolone, 5 days of plasmapheresis, and 5 days of immunoglobulin administered intravenously. She continued to have features of orofacial dyskinesia (that is, jaw opening and closing, chewing, facial grimacing, lip pouting) and tongue protrusion, which are characteristic features of this illness with autonomic instability; she remained intubated.

During the search for an ovarian teratoma, an MRI of her abdomen and pelvis showed a left ovarian 2.6 cm simple cyst and a right ovarian 19 mm hemorrhagic cyst with no evidence of a teratoma. A whole body positron emission tomography (PET) scan was negative for malignancy. The serum tumor markers carcinoembryonic antigen (CEA) and CA-125 were also negative.

**Fig. 2:**
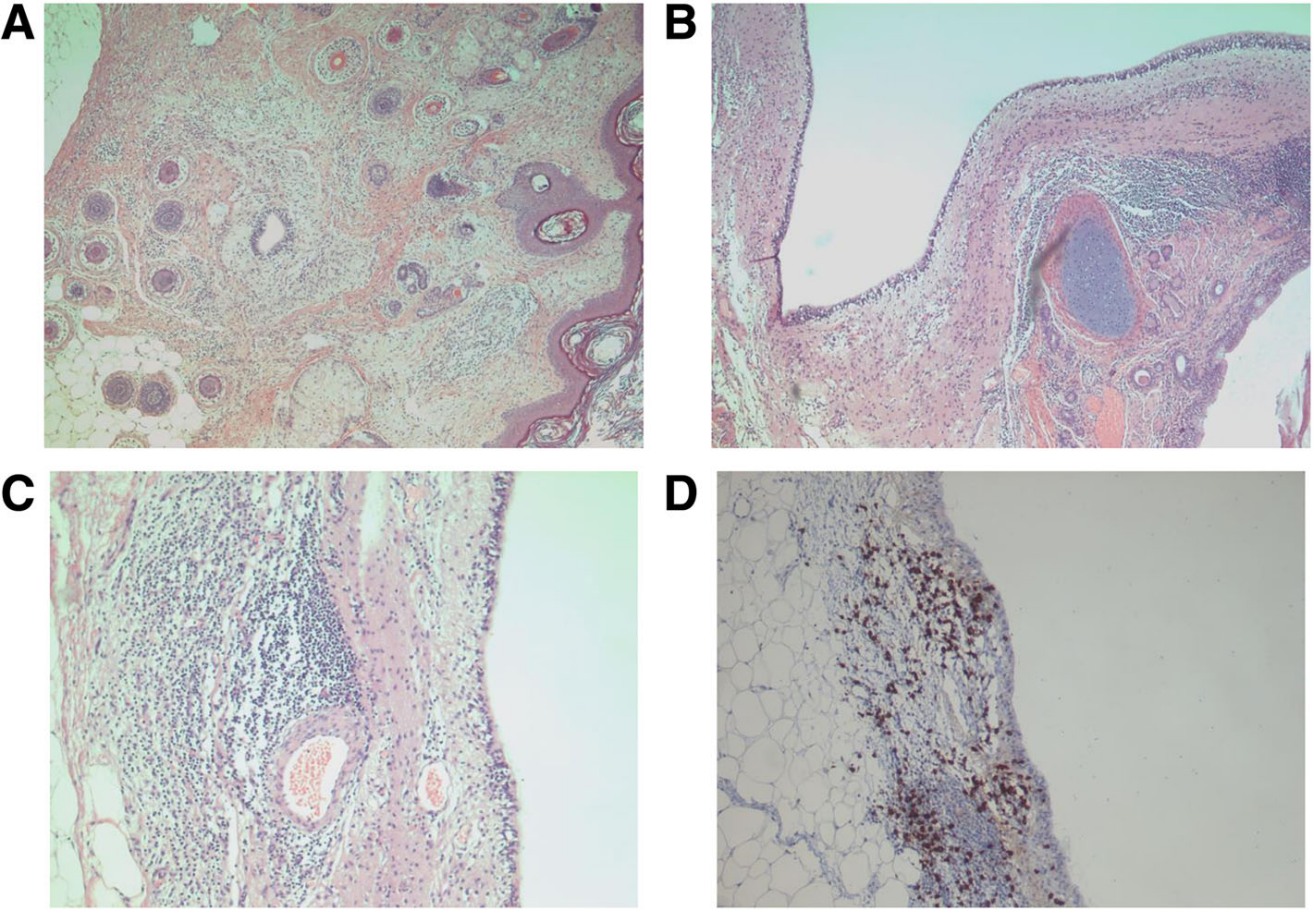
Histopathology of the left ovary. **a** Wall of cyst with an ependymal-lined cavity with surrounding glial tissue, in turn flanked by lymphocytes. Squamous epithelium to the *right*. Hematoxylin and eosin, original magnification × 50. **b** Broad cavity lined by ependyma, recapitulating a ventricular structure. Lymphocytes present to *lower right*. Bronchial structures also present. Hematoxylin and eosin, original magnification × 50. **c** Higher magnification demonstrates close relationship of lymphocytes to glial tissue in the subependymal-like neural structures. Hematoxylin and eosin, original magnification × 100. **d** Immunohistochemistry for CD8 demonstrates cytotoxic T lymphocytes. CD4-positive T helper cells and CD20-positive B lymphocytes were also present. Original magnification × 100

A multidisciplinary team discussed with her family the risks and benefits of bilateral oophorectomy including premature menopause, the need for hormone replacement therapy (HRT), and fertility issues balanced with the chances of neurological recovery. A decision was made to proceed with laparoscopic bilateral salpingo-oophorectomy and harvesting of ovarian tissue for cryopreservation, despite no convincing radiological evidence of an ovarian teratoma; the procedure was performed 29 days after admission. Histopathology of her left ovary revealed a mature cystic teratoma/dermoid cyst with mature neuroglial elements resembling a cerebral ventricle (Fig. 2). The ovarian teratoma had a prominent inflammatory response associated with the neural/glial elements: cytotoxic T lymphocytes (CD8-positive cells) were prominent; CD4-positive T helper cells and CD20-positive B lymphocytes were also found. This inflammatory response is unusual for teratomas, and points to the cellular immune response involved in our patient’s encephalitis.

Postoperatively she continued to receive rituximab followed by cyclophosphamide. She started to show signs of improvement with fluctuating level of consciousness: intermittently obeying commands, spontaneous eye opening, mouthing words, and smiling. She suddenly improved and a repeat CT of her head showed some volume loss of her right hippocampus.

One month later she was oriented with a Glasgow Coma Scale of 15/15 and was transferred to our medical ward where she remained seizure free on levetiracetam. A cognitive screen showed no deficits with a Mini–Mental State Examination (MMSE) score of 26/30, a repeat EEG showed a major reduction in the severity of the slow wave change and the disappearance of epileptogenic activity. A follow-up brain MRI showed developing hippocampal atrophy on the right.

At 3 months, our patient maintained an excellent recovery, apart from mild short-term memory impairment. She regained strength and was mobilizing independently with no further involuntary movements or seizures. She remained well at 7 months post-diagnosis and was fully independent in all acts of daily living, with some symptomatic improvement in memory, such that her MMSE was 30/30 and her Addenbrooke’s Cognitive Examination score was 98/100. Her EEG had normalized and MRI revealed persistent involutional change to the right hippocampal head. The NMDAR antibodies remained positive in her blood, one year after recovery.

## Discussion

This case is typical of anti-NMDAR encephalitis in that it involved a young woman with prodromal features followed by seizures, agitation, behavioral changes, coma, and autonomic instability. The evolving clinical features despite acyclovir therapy, plus the MRI appearance of bilateral limbic encephalitis led to the search for non-infective etiologies. In a cohort study by Chow *et al.* on the use of clinical and radiological features to distinguish temporal lobe HSV encephalitis from its mimics, autoimmune encephalitis was more frequently associated with psychotic symptoms, less fever at presentation, and the involvement of the bilateral temporal lobes on MRI when compared to HSV encephalitis [4].

The diagnosis of anti-NMDAR encephalitis in this case led to the search for an ovarian teratoma. Repeated imaging investigations showed no convincing evidence of an ovarian teratoma, however, the persistent coma and autonomic instability, despite aggressive immunotherapy, left the treating team in an ethical dilemma, that is to proceed with blind bilateral oophorectomy in a 25-year-old woman with no imaging evidence of teratoma, versus a watch and wait approach keeping in mind that delayed treatment is associated with a worse neurological outcome. It has been reported in the literature that without surgical intervention or with late teratoma removal, the illness might be prolonged or even lethal [3]. Having said that, and given that our patient’s age fell in the teratoma-associated anti-NMDAR encephalitis range, the benefits of oophorectomy were justified.

A cohort study by Titulaer *et al*. of 577 patients with anti-NMDAR encephalitis showed that the highest frequency of teratoma association with anti-NMDAR encephalitis was among the 12 to 45-year-old age group, with 52 % in females older than 12 years [13]. Our identification of the microscopic teratoma and our patient’s positive clinical response following the operation was rewarding to the patient, her family, and the treating team. Our experience has also been confirmed by others. For example, Boeck *et al*. reported the case of a 34-year-old woman with anti-NMDAR encephalitis who underwent bilateral oophorectomy, despite negative imaging results, after 11 months in an ICU; the histology confirmed a teratoma and it was only after the removal of the teratoma that the patient began to recover [14]. A 35-year-old woman had a blind bilateral oophorectomy following a 6-month history of refractory generalized status epilepticus secondary to anti-NMDAR encephalitis; the pathology revealed a microscopic teratoma and its removal was associated with significant neurological improvement [15].

Although there have been reports of delayed tumor removal leading to death, there have been other patients with recovery following immunotherapy alone when no tumor was detected [12]. The literature remains controversial regarding oophorectomy, when no convincing imaging evidence exists, to increase the likelihood of identifying a microscopic teratoma [12]. Some advocate treating only with aggressive immunotherapy and periodic screening for a tumor [16]; others recommend using all immunotherapeutic protocols before proceeding to bilateral oophorectomy in refractory patients [12].

## Conclusions

The neurological severity of anti-NMDAR encephalitis, the potential for a fatal outcome, and the proven benefits of early teratoma removal should promote a high index of suspicion for microscopic teratoma contributing to the patient’s neurological state, if proven refractory to immunotherapy.

## Abbreviations

CEA: Carcinoembryonic antigen (serum tumor marker)
CSF: Cerebrospinal fluids
CT: Computed tomography
EEG: Electroencephalogram
HRT: Hormone replacement therapy
HSE: Herpes simplex encephalitis
HSV: Herpes simplex virus
ICU: Intensive care unit
MMSE: Mini–Mental State Examination
MRI: Magnetic resonance imaging
NMDAR: N-methyl-D-aspartate receptor
PET: Positron emission tomography
